# Putting People First: A Primary Health Care Success in Rural India

**DOI:** 10.4103/0970-0218.66896

**Published:** 2010-04

**Authors:** Carol Vlassoff, Marcel Tanner, Mitchell Weiss, Shobha Rao

**Affiliations:** Department of Epidemiology and Community Medicine, University of Ottawa, Ottawa, Canada; 1 Swiss Tropical and Public Health Institute, University of Basel, Basel, Switzerland; 2 Agharkar Research Institute, Pune, Maharashtra, India

**Keywords:** Longitudinal perspective, Maharashtra, primary health care, reforms, three decades, WHO

## Abstract

**Background:**

The World Health Report, 2008, contains a global review of primary health care on the 30th anniversary of the Declaration of Alma-Ata. The period covered by the study reported on here corresponds with that of the Report, allowing for a comparison of achievements and challenges in one primary health care centre vis-a-vis the WHO standards.

**Materials and Methods:**

This study uses qualitative and quantitative data from a rural primary care facility in Western Maharashtra, collected over three decades. It analyzes the four groups of reforms defined by WHO in the context of the achievements and challenges of the study facility.

**Results:**

According to the WHO Report, health systems in developing countries have not responded adequately to people’s needs. However, our in-depth observations revealed substantial progress in several areas, including in family planning, safe deliveries, immunization and health promotion. Satisfaction with services in the study area was high.

**Conclusion:**

Adequate primary health care is possible, even when all recommended WHO reforms are not fully in place.

## Introduction

According to the World Health Report 2008, *Primary Health Care, Now More than Ever,*(1) health systems in developing countries have not responded adequately to people’s needs. The report argues that health systems are failing because they have not kept abreast of the challenges of a changing world. It suggests that health authorities have tended to view primary health care, not as a key set of reforms, but as one health care model among others. On the basis of the spirit of ”health for all”, lessons learned from experience and today’s health challenges, the report specifies four groups of reforms required to deliver quality primary health care services:

- Universal coverage to improve health equity,
- People-centered service delivery,
- Public policy reforms to promote and protect community health, and
- Leadership reforms to make health authorities more reliable.

The report argues that people in resource-constrained settings should not have to settle for less than the full provision of these reforms:

…it is not sufficient to respond to people’s desires to live in conditions that protect their health, support health equity and enable them to lead the lives that they value. People also expect their governments to put into place an array of public policies to deal with health challenges, such as those posed by urbanization, climate change, gender discrimination or social stratification.(2)

The period of the WHO review corresponds to that of a longitudinal study in Western Maharashtra, where the principal author conducted three studies on social, economic and health characteristics of one rural community – in 1975-1976, 1987 and 2007-2008. We are thus able to compare the global findings and recommendations of the WHO Report with our in-depth observations of primary health care in the study area over the same period.

### The primary health center

Long before the Declaration of Alma Ata, India adopted a primary health care model based on the principle that inability to pay should not prevent people from accessing health services. Derived from the recommendations of the Health Survey and Development Committee Report (the ”Sir Joseph Bhore Committee Report”) of 1946, the Indian Government resolved to concentrate services on rural people.(3) With programs such as the national family planning program, launched in 1952, and the policy of one community health worker per 1,000 people in the 1970s, India had already committed to most of the Alma Ata principles when the global primary health care movement began.

In 2005, the United Progressive Alliance Government launched the National Rural Health Mission (NRHM) to improve access to quality health care, especially for poor rural women and children, to strengthen primary health care institutions, increase equity and the decentralization of services, and encourage states to generate alternate sources of financing. While the Mission covers the entire country, its emphasis is on 18 states with the poorest infrastructure. Maharashtra, although not one of the focus states, does receive limited support from the NRHM.

The primary health center (PHC) described here is located in Limb, a community of about 10,000 people in Satara district. The area is heavily dependent on cash crops such as sugarcane, vegetables and flowers. Established as a fledgling health outpost in the mid-1970s, the PHC became a sub-center responsible for five communities in the early 1980s, and was upgraded to a full center in 1989, serving 20 villages and a population of 47,000. The study community, mentioned above, with a population of 3,326, is one of those served by the PHC. The facility is staffed by two medical officers, one of whom has been there since 1991, and the other, since 2005. In 2008 it had four health assistants, eight auxiliary nurse midwives, five multi-purpose workers, one pharmacist, one laboratory technician, four supervisors and seven support staff.

## Materials and Methods

The study used both qualitative and quantitative interview methods. Informed consent was obtained from the respondents, based on ethical consent forms. Qualitative information was collected through in-depth interviews of key health personnel, including the Satara District Health Officer (DHO), the Civil Surgeon, and several of their staff. The DHO, located in the Satara district headquarters, is responsible for oversight of the implementation of preventive and other primary health care activities in rural hospitals and primary health care centers. The Civil Surgeon’s office, in Satara’s Civic Hospital, oversees curative and clinical care at urban and rural hospitals. At the Limb PHC, the two senior medical officers were interviewed on four occasions, as were most other staff. The interviews lasted approximately two and three hours each, in Satara and Limb, respectively. Five private doctors from surrounding communities were also interviewed concerning their perceptions of the quality and services of the Limb PHC.

Quantitative data on clients’ use and satisfaction with PHC services were obtained from questionnaires with 494 married women, aged 15-49, from the study community, located about 1 km from the PHC. These questions formed part of a longer interview schedule on social, economic and health issues, mentioned above. In the first study, the principal author conducted all interviews personally; in the subsequent studies, two female assistants with social science backgrounds administered the women’s questionnaires. All qualitative information was collected by the principal author personally.

## Results

### Progress in study area

Over the past 30 years, progress in the health of the population served by the PHC was noted in several areas: lower disease rates due to better sanitation, including waste disposal and treatment of drinking water; improved hygiene as a result of increased awareness and health education; and reductions in dental problems, skin diseases and parasitic infections. These changes were attributable to collaboration between the health and education sectors, media information and efforts of village leaders to improve sanitation. School programs provide nutrients, worm medicines, hemoglobin tablets and folic acid tablets to students, and teachers assist with immunizations and health promotion.

### WHO reforms and the PHC

The first WHO reform, universal coverage to improve health equity, refers to ”universal access to the full range of personal and non-personal health services they [people] need, with social health protection”,(4) addressing social and economic inequities as well as health.

The PHC services are equitable in the sense that their main users are women and children, the less advantaged members of rural society, and in terms of the range of services provided, including those considered as essential for primary health care.(5) These comprise emergency services, maternal and child health, family planning, immunization, school check-ups and the detection of infectious diseases.

The 494 women interviewed said they relied on the PHC for family planning, deliveries, vaccination, sanitation and health promotion, while mostly visiting private doctors for other services. Satisfaction with the PHC was extremely high. Of the 471 women who rated the PHC*, 88% rated the services as good.** Women were also asked if they thought the services of the PHC had improved since it had been upgraded to a full center. Of the 343 women who replied***, 72% said that they had improved significantly, mentioning the availability of beds (now 10 compared to only five in 1989), an operating theater, the accessibility of essential medicines, the facility’s cleanliness and spaciousness, and on-site water for drinking and bathing.

Longitudinal data from the married female respondents indicated that the PHC services were having a positive effect. Average fertility declined from 3.1 live births and 2.7 living children in 1975-1976 to 2.3 live births and 2.1 living children in 2007-2008. Sterilization increased from 29% in 1975-1976 to 71% in 2007-2008. Contraceptive use grew from almost total reliance on sterilization to a diversity of methods, including the IUD, pill and condoms, used by 11%, 16% and 21% of the respondents, respectively. Employing contraception for spacing, an important health intervention for women and children, was virtually unheard of in the area 30 years ago. Another indicator of PHC program success was a decline in infant and child deaths in the study community. Of the 1975-1976 respondents, 19% had experienced an infant death compared to only 9% of the respondents in 2007-2008. Similarly, 25% of the 1975-1976 respondents had lost at least one child, compared to only 10% in 2007-2008.

The PHC services do not directly address issues of social and economic inequities. Nonetheless, all essential medicines and family planning commodities are provided free of charge. Although clients are encouraged to give small voluntary donations to the clinic, few do so.

The second WHO reform, people-centered health systems, focuses on clients’ broad health needs, including enduring personal relationships, comprehensive, continuous and person-centered care, health of all community members, tackling health determinants, and involving people in their own health care and that of their community.

Our findings indicate that people-centeredness is the greatest achievement of the Limb PHC. Unlike many clinics that fail to consider their clients’ time availability,(7) the PHC opens in the mornings and evenings seven days a week, with full services on Sundays to accommodate weekly market visitors. The staff were popular with their clients, demonstrated by the fact that the community put pressure on district authorities to retain the head medical officer for a second term, contrary to the normal practice of rotation of every 10 years. Nonetheless, and as has been observed elsewhere,(8) provider-client relationships tended to be top-down: patients were not encouraged to engage in dialogue with health workers. Interestingly, patients considered this vertical relationship to be entirely normal and said they did not expect to be asked for their input.

According to WHO, people-centeredness requires regular visits of PHC staff members to defined populations to whom they are assigned. This is true of study area where malaria workers made fortnightly visits to households, taking blood smears, providing education and conducting container surveys. Immunization workers also visited people’s homes to distribute information about health campaigns, such as vaccination days. The PHC staff members reported satisfaction with their regular community visits, facilitating interaction with the population and helping them assess the quality of their services. Generally, satisfaction was high among PHC personnel.

The third type of reform highlights public policies to promote and protect health. In the PHC, the policy environment focuses mainly on family planning, rather than on a more comprehensive approach to reproductive health. This emphasis has not changed in 30 years, despite significant fertility declines and low fertility levels in the PHC area (Crude Birth Rate=16.7). Incentives for health workers are still used to promote the 1-2 child policy, and district officers are striving to meet the 2010 target of 15 births per 1,000 population.

In recent years, the PHC has benefited from NRHM policies and programs: public expenditure on health has more than doubled. The annual government grant per medical officer increased from Rs. 75,000 in 2007 to Rs. 175,000 in 2008, and for the first time, assistant nurse midwives received small discretionary budgets for mother and child health activities.

The fourth WHO reform emphasizes leadership within and beyond the health sector. It ask governments to be brokers for PHC reform, to mediate ”the social contract for health”, promote participation, negotiation and policy dialogue. This requires strong information systems for planning and programming, and for harnessing innovations and lessons learned.

The Limb PHC is recognized throughout the area for its leadership. For example, during the study period, it was selected to host many training programs for students from surrounding areas. Nonetheless, much more financial and technical support would be required for it to comply with the fourth WHO reform. There is a considerable potential for greater *panchayat* involvement in, and financing of, PHC activities. A study in Kerala found, for example, that a modest increase of 2% to PHC budgets from local governments resulted in major improvements in patient coverage, cost-effectiveness, medicine supply, information and patient satisfaction.(9)

The rather hierarchical provider-patient relationship, while both accepted and sanctioned in the community, limits the potential for patient participation, negotiation and policy dialogue. Clients could perhaps benefit from more involvement, although, as rural cultivators, they have limited time for ongoing input.

The collection and use of data for planning and patient follow-up is a weakness of the PHC, as elsewhere in India.(10) One medical officer reported that efforts are made to collect and record client information: patients are given number cards and advised to bring them to the PHC whenever they visit. However, the other officer was less positive about this system. ”No one pays any attention to them,” he said. Gathering data is seen as one more chore in workers’ daily routines, not as a vital component of PHC activities.

## Discussion

Our findings support the argument of Lawn *et al*. that ”there are encouraging signs at all levels of a shift toward embracing a more comprehensive menu of health intervention content and a more comprehensive health system building, if not yet a major shift toward a fully participatory, comprehensive process.”(5) Although far from perfect, the study PHC demonstrated that exemplary leadership, a sound understanding of local issues and a focus on ”the centrality of people in public health and its practice”(11) can provide competent and adequate services. People in the study area did not expect the health system to provide an enabling social and normative environment as prescribed by the WHO. They were content to have their basic health needs addressed.

On the basis of our findings, a backbone of user and provider satisfaction is crucial, based on a well trained work force that demonstrates leadership and client-centeredness. Adequate financing, albeit modest, must be available for essential services, supplies and medicines. In the study area, there is potential for greater involvement of local *panchayats* and perhaps for enforcing a small user fee. These could represent significant resources for health, as noted elsewhere in India.(12) Smoothly functioning logistical and supply systems must also be in place to reach outlying communities.

Implicit in these requirements are policies of universal coverage and equity, but these policies, and strategies for their implementation, may not be explicitly or fully enunciated. To monitor the impact of primary health care, health information systems must be developed. The widespread availability of information technology should be leveraged by the primary health care system in far more creative ways.(13,14)

A major challenge for the future, as others have also noted,(15,16) is the re-evaluation of primary health care priorities in the light of India’s ageing population and the growing prevalence of non-communicable diseases. The potential for people’s participation in the process of re-evaluation of health priorities is substantial, a potential that remains largely unexploited by the health system.

We conclude that our appreciation of modest, tangible achievements, such as those observed in the study area, should not be eclipsed by focusing on ideal scenarios, such as those set out in the World Health Report. While primary health care should always aspire to higher goals, it needs to take a gradual, incremental approach.

## Footnotes

* Some of the women had not used the PHC because they had only been in the village for a short time.

** Kumar Patro et al, also found high levels of satisfaction in a New Delhi PHC, despite weaknesses in the services observed by the researchers.(6)

*** Many of the younger married women had only been in the village a short time and could not compare the two periods.

## References

[1] (2008). World Health Organization (WHO). World Health Report 2008.

[2] (2008). WHO. World Health Report 2008.

[3] Goel S (2008). From Bhore committee to national rural health mission: A critical review. Int J Health.

[4] (2008). WHO. World Health Report 2008.

[5] Lawn JE, Rohde J, Rifkin S, Were M, Paul VK, Chopra M (2008). Alma-Ata 30 years on: Revolutionary, relevant, and time to revitalize. Lancet.

[6] Patro BK, Kumar R, Goswami A, Nongkynrih B, Pandav CS; UG Study Group (2008). Community perception and client satisfaction about the primary health care services in an urban resettlement colony of New Delhi. Indian J Community Med.

[7] Kumari R, Idris M, Bhushan V, Khanna A, Agarwal M, Singh S (2009). Study on patient satisfaction in the government allopathic health facilities of Lucknow district, India. Indian J Community Med.

[8] Ganguly E, Deshmukh P, Garg B (2008). Quality assessment of private practitioners in rural Wardha, Maharashtra. Indian J Community Med.

[9] Varatharajan D, Thankappan R, Jayapalan S (2004). Assessing the performance of primary health centres under decentralized government in Kerala, India. Health Policy Plan.

[10] Sharma AK (2009). National rural health mission: Time to take stock. Indian J Community Med.

[11] Macfarlane S, Racelis M, Muli-Musiime F (2000). Public health in developing countries. Lancet.

[12] Dipankar Rao K, Peters DH (2007). Quality improvement and its impact on the use and equality of outpatient health services in India. Health Econ.

[13] Dasgupta A, Deb S (2008). Telemedicine: A new horizon in public health in India. Indian J Community Med.

[14] Peters DH, Kohli M, Mascarenhas M, Rao K (2006). Can computers improve patient care by primary health care workers in India?. Int J Qual Health Care.

[15] Srinath Reddy K, Shah B, Varghese C, Ramadoss A (2005). Responding to the threat of chronic diseases in India. Lancet.

[16] Mendoza Aldana J, Piechulek H, al-Sabir A (2001). Client satisfaction and quality of health care in rural Bangladesh. Bull World Health Organ.

